# Supplementary material for: Comparative genomics provides new insights into the diversity, physiology, and sexuality of the only industrially exploited tremellomycete: Phaffia rhodozyma
Source: BMC Genomics. 2016 Nov 9;17:901. doi: 10.1186/s12864-016-3244-7 (PMC5103461; doi:10.1186/s12864-016-3244-7)
Supplement: Additional file 6: — List of orphan genes with links to PFAM (related to Additional file 1: Table S1). (ZIP 1428 kb) [file 12864_2016_3244_MOESM6_ESM.zip › PFAM_UNIQUE/G04332_P.html]

#                                                                            --- full sequence --- -------------- this domain -------------   hmm coord   ali coord   env coord
  
# target name        accession   tlen query name           accession   qlen   E-value  score  bias   #  of  c-Evalue  i-Evalue  score  bias  from    to  from    to  from    to  acc description of target
  
#------------------- ---------- ----- -------------------- ---------- ----- --------- ------ ----- --- --- --------- --------- ------ ----- ----- ----- ----- ----- ----- ----- ---- ---------------------
  
G04332\_P             -            387 ARGLU                PF15346.1    149     0.047   12.1   7.9   1   1   0.00026     0.099   11.1   7.9    48    97   296   345   286   353 0.86 - Arginine and glutamate-rich 1  
G04332\_P             -            387 Atrophin-1           PF03154.10   982      0.72    6.6   6.6   1   2       1.4   6.3e+02   -3.2   0.1   457   479    88   110    42   137 0.63 - Atrophin-1 family  
G04332\_P             -            387 Atrophin-1           PF03154.10   982      0.72    6.6   6.6   2   2   0.00019     0.088    9.6   2.6   572   626   288   354   252   373 0.58 - Atrophin-1 family  
G04332\_P             -            387 Caldesmon            PF02029.10   492       1.2    6.1   5.5   1   1     0.018       1.9    5.5   5.5    57   106   308   360   294   380 0.56 - Caldesmon  
G04332\_P             -            387 DUF4482              PF14818.1    141     0.033   13.5   1.1   1   1   7.7e-05     0.092   12.1   1.1    21    55   318   352   302   379 0.77 - Domain of unknown function (DUF4482)  
G04332\_P             -            387 ERM                  PF00769.14   246       1.1    7.4   4.4   1   1     0.012       1.8    6.7   4.4    78   144   302   366   290   386 0.59 - Ezrin/radixin/moesin family  
G04332\_P             -            387 HR1                  PF02185.11    70      0.36    9.3   2.7   1   1   0.00093       0.8    8.2   2.7    31    63   313   347   301   354 0.78 - Hr1 repeat  
G04332\_P             -            387 Jnk-SapK\_ap\_N        PF09744.4    158     0.048   12.3   2.3   1   1   4.1e-05     0.082   11.6   2.3    71   109   306   344   289   354 0.88 - JNK\_SAPK-associated protein-1  
G04332\_P             -            387 KfrA\_N               PF11740.3    120       2.6    7.2   5.5   1   1    0.0061       7.3    5.7   5.5    70   114   299   343   292   347 0.89 - Plasmid replication region DNA-binding N-term  
G04332\_P             -            387 MAP7                 PF05672.6    171      0.37    8.8   7.4   1   1    0.0029      0.72    7.9   7.4    39    73   309   342   292   378 0.57 - MAP7 (E-MAP-115) family  
G04332\_P             -            387 Myosin\_tail\_1        PF01576.14   859      0.59    6.4   3.3   1   1    0.0056      0.83    5.9   3.3   101   147   301   347   295   357 0.77 - Myosin tail  
G04332\_P             -            387 Radial\_spoke\_3       PF06098.6    291      0.74    7.4   5.3   1   1   0.00073       1.1    6.9   5.3   164   216   303   354   298   377 0.76 - Radial spoke protein 3  
G04332\_P             -            387 Tropomyosin          PF00261.15   237       3.8    5.2   6.0   1   1     0.026       6.3    4.5   6.0    82   123   303   341   298   353 0.83 - Tropomyosin
